# Adsorption of Butylparaben and Methylene Blue from Aqueous Solution Using Activated Carbon Derived from Oak Bark

**DOI:** 10.3390/ma18132984

**Published:** 2025-06-24

**Authors:** Dorota Paluch, Robert Wolski, Aleksandra Bazan-Wozniak, Robert Pietrzak

**Affiliations:** Department of Applied Chemistry, Faculty of Chemistry, Adam Mickiewicz University, Uniwersytetu Poznańskiego 8, 61-614 Poznan, Poland; dorota.paluch@amu.edu.pl (D.P.); robert.wolski@amu.edu.pl (R.W.); aleksandra.bazan@amu.edu.pl (A.B.-W.)

**Keywords:** activated carbon, oak bark, physical activation, butylparaben, methylene blue

## Abstract

This study presents the production of activated carbon through the direct physical activation of oak bark using carbon (IV) oxide. The activation process was conducted at three distinct temperatures of 700 °C, 800 °C, and 900 °C. The activation time was 60 min. A comprehensive series of analytical procedures was performed on the resultant adsorbents. These included elemental analysis, determination of textural parameters, Boehm titration, pH determination of aqueous extracts, pH_pzC0_, assessment of ash content, and elemental and XPS analysis. Subsequently, adsorption tests for butyl paraben and methylene blue were carried out on the materials obtained. The total surface area of the sorbents ranged from 247 m^2^/g to 696 m^2^/g. The acid-based properties of the samples tested were examined, and the results indicated that the sorbents exhibited a distinct alkaline surface character. The sorption capacities of the tested samples for butylparaben ranged between 20 and 154 mg/g, while the capacities for methylene blue varied between 13 and 224 mg/g. The constants of the Langmuir and Freundlich models were determined for each of the impurities, as well as the thermodynamic parameters. The present study investigates the influence of contact time between adsorbent and adsorbate, in addition to the kinetics of the adsorption processes. The activated carbon samples obtained demonstrated satisfactory sorption capacities, with the material obtained at 900 °C exhibiting the best sorption capacities.

## 1. Introduction

In recent decades, there has been an increasing focus on the effect of industrial chemicals on both the environment and human health. Of these chemicals, endocrine-disrupting chemicals (EDCs) are of particular concern. These substances have the capacity to disrupt the normal functioning of hormonal systems in animals and humans, even at very low concentrations [1]. EDCs are present in a wide range of everyday products, including plastics, cosmetics, pharmaceuticals, food packaging, and pesticides. These chemicals are released into the environment through domestic and industrial wastewater [2,3].

One extensively utilized group of EDCs is parabens, which are chemical preservatives frequently incorporated into personal care products, pharmaceuticals, and certain foods [4]. The presence of butylparaben (BuP) in various environmental matrices, including surface water, groundwater, sewage sludge, soil, and air samples, is of particular concern. Despite its low concentration, BuP can still have harmful effects over time. Research has demonstrated that it can induce oxidative stress, disrupt hormonal function, influence the behavior of aquatic organisms, and modify microbial communities [5]. This phenomenon may even contribute to the growing problem of antibiotic resistance. In human subjects, BuP has been detected in hair samples, indicating that exposure is widespread, primarily through cosmetics, contaminated water, air, and dust [6].

This mounting body of evidence has given rise to a heightened level of concern regarding the environmental presence and impact of BuP. As an ester of 4–hydroxybenzoic acid, BuP is chemically stable enough to persist in various ecosystems [5]. Concentrations of the substance have been reported in surface waters ranging from a few to several tens of nanograms per liter and in sewage sludge up to several thousand nanograms per kilogram [7,8]. These findings underscore the pervasive utilization of this substance and its substantial dispersal via municipal and industrial wastewater systems, thereby contributing to contamination in both aquatic and terrestrial environments.

The presence of such pollutants in water bodies has been increasing, and this highlights a major issue. Conventional wastewater treatment plants are not equipped to efficiently eliminate endocrine-disrupting chemicals like butylparaben [9]. In addition to EDCs, another class of persistent pollutants entering aquatic systems is synthetic dyes, notably methylene blue. Methylene blue is a toxic, stable dye that is used widely in the textile, paper, and chemical industries, though it is not classified as an EDC [10]. Its presence in wastewater is concerning due to its resistance to degradation, potential toxicity to aquatic life, and role as a model contaminant in testing water purification technologies. Therefore, the removal of pollutants like BuP and methylene blue is crucial to ensuring water safety and ecosystem protection.

Consequently, there is an increasing demand for more advanced, efficient, and affordable treatment methods to protect public health and the environment [11]. In the field of water treatment, adsorption has become the leading technology for eliminating trace organic pollutants. This efficacy is attributable to the multifaceted nature of adsorption, which encompasses a range of processes that facilitate the removal of these pollutants from water sources. This method is characterized by its simplicity, cost-effectiveness, and capacity to detect even low concentrations of noxious compounds [12].

Active carbon is one of the most promising and sustainable adsorbents. It is a carbon-rich material produced by heating organic matter, such as plant waste, in the presence of limited oxygen. There are three key factors that make activated carbon appealing for environmental applications. Firstly, its production requires minimal financial investment. Secondly, its utilization does not result in the release of significant quantities of carbon into the atmosphere, thereby ensuring that it is a sustainable solution. Thirdly, activated carbon can be derived from a wide variety of biomass sources, including agricultural and herbal residues [13]. The material’s porous structure, extensive surface area, and surface chemistry render it conducive to the absorption of contaminants such as butylparaben and methylene blue from water.

Lignin, a by-product of the paper industry, is an abundant and complex polymer that is ideal for sustainable applications. Its structure makes it a promising precursor for activated carbon. Lignin’s high fixed carbon content enhances the thermal stability and yield of this process. Unlike cellulose-rich biomass, lignin can better withstand activation [14]. The use of black liquor lignin was investigated, and activated carbon with a surface area ranging from 174 to 310 m^2^/g was obtained through physical steam activation [15]. Wang et al. developed a lignin-based porous carbon with a layered graphene-like structure through direct carbonization, which exhibited a specific surface area of 376 m^2^/g [16]. Despite the simplicity of the direct carbonization method, the resulting carbon structure was highly unstable and exhibited small porosity. Other studies have shown that the chemical activation of oak bark using KOH results in activated carbon with a surface area ranging from 1180 to 1821 m^2^/g [17]. These findings highlight lignin-based materials as a promising precursor for activated carbon production, offering a sustainable and eco-friendly alternative to conventional sources. In particular, the use of physical activation methods aligns with green chemistry principles and reduces the need for harsh chemicals. However, further research is needed to optimize synthesis conditions and improve the structural stability and porosity of the resulting carbon materials.

The use of activated carbon in the removal of parabens from aqueous solutions has been described in the literature. Recent studies have explored diverse adsorbents, including activated carbon, showing promising results. For instance, modified activated carbons obtained from African palm shells have demonstrated sorption capacities of 103–194 mg/g for methyl- and ethylparaben [18]. Conversely, the adsorption capacity of butylparaben for modified activated carbon from African palm shells ranged from 144 to 268 mg/g [19]. Butylparaben adsorption studies on coconut-based activated carbon revealed a sorption capacity of 7.52 mg/g [20]. Despite these advancements, challenges remain in scaling up processes and understanding adsorption mechanisms across different water matrices. Continued research is essential to develop sustainable and high-performance adsorbents.

The repercussions of unsustainable material development and inadequate resource management prompt the adoption of concepts such as circular economy within the field of waste management. In the industry, the pursuit of cost-effective and accessible materials, coupled with comprehensive life cycle assessments, has consistently driven efforts towards sustainability and the advancement of greener chemistry. This research provides a thorough examination of the production and physicochemical properties of activated carbons obtained via the direct activation of oak bark using carbon dioxide. The material used is a by-product of the herbal industry and represents an environmentally friendly way of producing carbon bioadsorbents. This study’s novel contribution is the optimization of activated carbon production using oak bark as a precursor, with the aim of creating cost-effective carbon adsorbents with effective sorption capabilities for water pollutants, particularly butylparaben and methylene blue.

## 2. Materials and Methods

### 2.1. Activated Carbon Preparation

**Figure 1:**
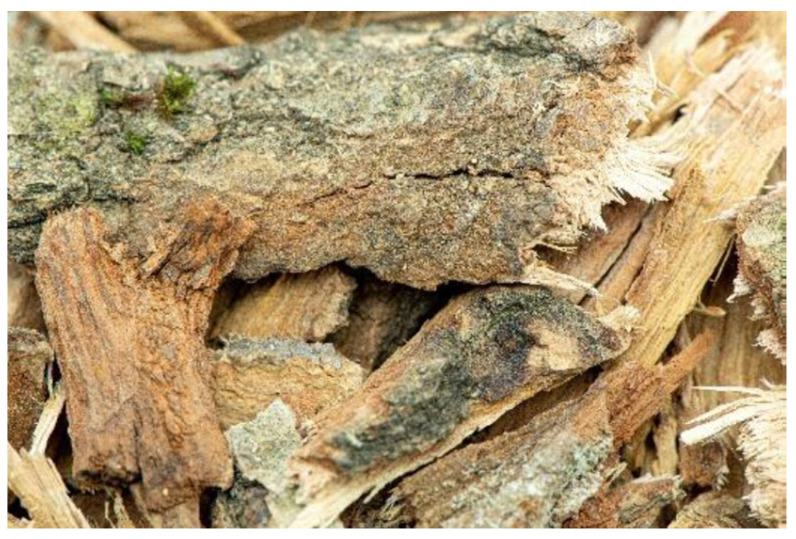
Oak bark.

The material used for the preparation of activated carbon samples was oak bark (*Quercus* L.) (Figure 1).

The precursor was ground into pieces measuring 2 mm by 3 mm. Then, the starting material was subjected to a drying process at a temperature of 110 °C for a duration of 12 h, after which it was divided into three distinct parts. The following three different temperatures were selected to directly activate the precursor: 700 °C (OB_DA7), 800 °C (OB_DA8), and 900 °C (OB_DA9). The activator employed was carbon dioxide, with a flow rate of 250 mL/min. The time of activation was 60 min, and the heating rate was 10 °C/min. The samples were cooled in a carbon dioxide atmosphere. Each experiment was conducted within a horizontal furnace, with each experiment undergoing replication thrice. The materials were subjected to a drying process until a solid mass was obtained. Thereafter, the materials were sieved through a 0.09 mm sieve and homogenized.

### 2.2. Characterisation of Activated Carbon

Textural parameters were determined by nitrogen (N_2_) adsorption–desorption isotherms measured at 77 K. The Brunauer–Emmett–Teller equation was used to ascertain the specific surface area. The amount of nitrogen adsorbed at a relative pressure of approximately 0.99 was taken into account to estimate the total pore volume. The volume and surface area of the micropores were determined using the t-plot method.

Determination of the iodine number was ascertained in accordance with the American Society for Testing and Materials (ASTM) method D4607-94.

The number of oxygen groups on the surface of the tested samples was determined using the Boehm method. Titration was performed using NaOH and HCl solutions of 0.1 mol/L. Three measurements were taken for each sample.

The pH_pzc_ of the activated carbon samples was determined. To achieve this, 0.1 M NaCl solutions with a fixed pH value between 2 and 12 were prepared. NaOH and HCl solutions were then used to adjust the pH. A quantity of 0.1 g of the prepared carbon samples was added to the solutions, after which the solutions were shaken at a rate of 300 rpm. Then, the final pH of each solution was measured.

The Thermo Scientific Flash 2000 Elemental Analyzer (Elementar Analysensysteme GmbH, Langenselbold, Germany). was utilized in order to ascertain the elemental composition of the precursor and activated carbons. Standard ash analysis was performed in accordance with ASTM D2866–94 (2004) standard.

### 2.3. Adsorption Studies

The model contaminants employed for the adsorption tests of the activated carbon samples were butylparaben and methylene blue (Table 1).

Samples of the activated carbon (20 mg) were introduced into aqueous solutions of the respective contaminants. The volumes of each solution were 0.05 L, and the concentrations of butylparaben ranged from 10 to 100 mg/L, while the concentrations of methylene blue ranged from 5 to 110 mg/L. The mixtures were then shaken at 200 rpm/min on a laboratory shaker at room temperature (295.15 ± 1 K). After 24 h, samples were taken. For the butylparaben solutions, the samples were filtered through a 0.22 μm syringe filter. The butylparaben content was analyzed using a Waters 2690 HPLC chromatograph equipped with a Waters 2487 Dual λ Absorbance Detector, Waters Corporation, Milford, MA, USA. Butylparaben content was analyzed at λ = 254 nm. The mobile phase consisted of a mixture of water and acetonitrile (63:37 *v*/*v*), with a flow rate of 0.8 mL/min and an injection volume of 5 μL. The limit of detection (LOD) with relative standard deviation (RSD) was found to be 0.252–0.580 μg/L ± 2.5–4.8%, while the limit of quantification (LOQ) with RSD was determined to be 0.831–1.914 μg/L ± 2.5–4.8%. Samples containing methylene blue were subjected to centrifugation using a laboratory centrifuge. The concentration of methylene blue in the collected samples was measured spectrophotometrically at a wavelength of λ = 665 nm using a Varian Carry Bio 100 dual-beam UV–VIS spectrophotometer, Thermo Fisher Scientific, Scoresby, Australia. These methods were employed for all adsorption studies. The following formula was applied to calculate the adsorption capacity of the adsorbents:

(1) $$q_{e}=\frac{C_{0}-C_{e}}{m}\times V$$

where C_0_ denotes the initial concentrations (mg/L) of the dye in the solution; C_e_ denotes the equilibrium concentrations (mg/L) of the dye in the solution; m denotes the mass of the activated carbon (g); and V denotes the volume of the solution (L).

In order to determine a suitable model for the adsorption on the obtained materials, the linear forms of the Langmuir and Freundlich equations were applied. The following linear equation describes the Langmuir isotherm (2):

(2) $$\frac{1}{q_{e}}=\frac{1}{q_{max}}+\frac{1}{K_{L}q_{m}}\times \frac{1}{C_{e}}$$

where q_e_ is the equilibrium amount of adsorbed substance (mg/g), K_L_ is the Langmuir equilibrium constant (L/mg), and q_max_ is the maximum adsorption capacity of the adsorbent (mg/g).

The following linear equation describes the Freundlich isotherm (3):

(3) $${logq}_{e}={logK}_{F}+\frac{1}{n}{logC}_{e}$$

where K_F_ is the Freundlich equilibrium constant (mg/g(L/mg)^1/n^), and 1/n is the adsorption intensity constant.

The initial butylparaben concentrations for the individual activated carbon samples were determined by estimating their adsorption capacities. For samples OB_DA7 and OB_DA8, the concentration was 10 mg/L, whereas for OB_DA9, it was 30 mg/L. However, for further research, the initial concentration of methylene blue was 10 mg/L for OB_DA7, 25 mg/L for OB_DA8 and 90 mg/L for OB_DA9.

This study was conducted to ascertain the effect of temperature on the adsorption of butylparaben and methylene blue from aqueous solutions. A 20 mg sample of activated carbon was added to 0.05 L of aqueous solutions of the organic pollutant. The samples were subjected to a 24-h shaking process at temperatures of 298.15 K, 308.15 K, and 318.15 K, with a shaking rate of 250 rpm/min. Subsequently, samples were collected for analysis, and the concentrations of butylparaben and methylene blue were determined.

The following formulas were used to calculate the thermodynamic parameters:

(4) $$∆G^{0}=-RTlnK_{d}$$

(5) $$∆G^{0}=∆H^{0}-T∆S^{0}$$

(6) $$lnK_{d}=\frac{∆S^{0}}{R}+\frac{∆H^{0}}{RT}$$

where ΔG^0^ denotes Gibbs free energy, R is the universal constant (8.314 J/mol × K), T is temperature (K), ΔH^0^ is the enthalpy change, ΔS^0^ is the entropy change, and K_d_ is the thermodynamic equilibrium constant.

The effect of the pH value of the aqueous solutions of butylparaben and methylene blue on the resulting activated carbons’ sorption capacity was determined. Solutions of butylparaben and methylene blue with fixed pH values (pH 3–11) were prepared. The pH of the solutions was adjusted using 0.1 M HCl or NaOH solutions.

In order to characterize the kinetics of the adsorption process, 20 mg of activated carbon samples were flooded with solutions of equal concentration of either butylparaben or methylene blue. The bottle was placed on a laboratory shaker at 300 rpm/min. The spectrophotometric measurements were taken over a period of 180 min for butylparaben and 300 min for methylene blue. The concentrations of butylparaben and methylene blue were determined according to the previously described procedure. The following two models were used for the purpose of data analysis: the pseudo-first-order model (7), the pseudo-second-order model (8), and intraparticle diffusion (9), as follows:

(7) $$\log(q_{e}-q_{t})=logq_{e}-\frac{k_{1}}{2.303}t$$

(8) $$\frac{t}{q_{t}}=\frac{1}{k_{2}q_{e^{2}}}+\frac{t}{q_{e}}$$

(9) $$q_{t}=k_{id}t^{1/2}+C$$

where q_e_ is the equilibrium amount of adsorbed substance (mg/g); q_t_ is the amount of adsorbed dye over time (mg/g); t is the process time (min); k_1_ is the pseudo-first-order adsorption constant (1/min); k_2_ is the pseudo-second-order adsorption constant (g/mg × min); k_id_ is the intraparticle diffusion constant (mg/g × min^1/2^); and C is the boundary layer constant (mg/g).

## 3. Results and Discussion

### 3.1. Physiochemical Characterization of the Activated Carbon

Table 2 presents the physicochemical properties of the activated carbon, including surface area, pore volume, pore size, and iodine number, which show a strong dependency on activation temperature. As demonstrated in the tabular data, there was a substantial increase in the total surface area, from 247 m^2^/g at 700 °C to 696 m^2^/g at 900 °C. This increase is accompanied by a progressive increase in total pore volume from 0.480 cm^3^/g to 1.445 cm^3^/g and in average pore size from 3.80 nm to 6.35 nm. These trends indicate increased pore development and structural expansion with higher thermal treatment, probably due to the increased release of volatiles and widening of existing pores. Interestingly, although the total surface area increases with temperature, the micropore surface area peaks at 800 °C (198 m^2^/g) and then decreases at 900 °C (141 m^2^/g). A similar trend is observed for the micropore volume. This suggests that, while higher temperatures promote mesopore formation and overall porosity, they may cause micropores to collapse or coalesce, resulting in a reduced microporous structure at 900 °C. Iodine number, a common indicator of microporosity and adsorption capacity for small molecules, shows a significant increase with temperature from 277 mg/g at 700 °C to 741 mg/g at 900 °C. Despite the decrease in micropore volume at 900 °C, the increase in iodine number indicates the presence of a well-developed accessible surface area and a wider pore size distribution that facilitates iodine uptake. The yields of the activated carbon samples obtained ranged from 38.91% to 49.24%. Overall, the data show that increasing the activation temperature improves the textural properties of activated carbon, in particular favoring the development of mesopores, which is beneficial for the adsorption of larger molecules such as methylene blue. The 900 °C sample, with the highest surface area, pore volume, and iodine number, exhibits the most favorable properties for adsorption applications.

**Figure 2:**
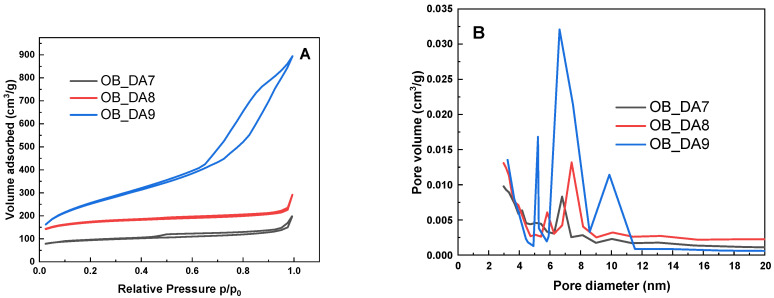
Low-temperature N_2_ adsorption–desorption isotherms (**A**) and pore size distribution (**B**) of the activated carbons obtained.

As illustrated in Figure 2, the low-temperature nitrogen adsorption/desorption isotherms (A) and pore distribution (B) for the activated carbons under investigation are presented. According to the International Union of Pure and Applied Chemistry (IUPAC) classification [21], these isotherms can be assigned to type IV(a). Type IV(a) adsorption isotherms are a characteristic feature of mesoporous materials. This isotherm is distinguished by the occurrence of capillary condensation within pores with diameters that fall within the mesopore range (from 2 to 50 nm). The shape of the curves is confirmed by the data summarized in Table 2. According to the IUPAC classification, six distinct types of hysteresis loops have been identified, which are closely correlated with the characteristics of the pore structure and the adsorption mechanism. The hysteresis loops exhibited in the graph are classified as type H4 for all samples, which is indicative of materials characterized by narrow, gaping pores [21]. According to the IUPAC classification, type H4 is defined as a hysteresis known as “ink-bottle” hysteresis. This phenomenon of hysteresis is a distinctive attribute of materials that exhibit a transition from narrow apertures to significantly broader spaces.

The activated carbon samples produced in this study exhibit significantly improved textural properties compared to those reported for fennel seed [22]. Activated carbon samples produced from fennel seed and oak bark by direct physical activation with CO_2_ exhibit markedly different textural and adsorptive properties. At comparable activation temperatures, oak bark consistently outperforms fennel seed in terms of surface area, pore volume, and iodine number. Specifically, at 800 °C, the surface area of oak bark-based carbon reached 452 m^2^/g, whereas fennel seed-based carbon reached only 14.6 m^2^/g. This trend continued at higher temperatures, such as when oak bark carbon reached 696 m^2^/g at 900 °C, demonstrating a significant development of porous structure with increasing temperature, whereas fennel seed showed limited activation even at 800 °C. Pore volume data further emphasize this difference. Oak bark adsorbents exhibited total pore volumes of 0.958 cm^3^/g at 800 °C and 1.445 cm^3^/g at 900 °C, compared to only 0.09 cm^3^/g for fennel seed at the highest temperature tested. In addition, the average pore size of oak bark carbon remained in the microporous range (approximately 4.81–6.35 nm), whereas fennel seed samples developed much larger pores (25.75–53.01 nm), indicating less efficient pore development. This difference in pore structure is reflected in the iodine values, as oak bark samples reached up to 741 mg/g at 900 °C, whereas fennel seed samples peaked at only 246 mg/g. The significantly higher iodine values of oak bark suggest a higher proportion of micropores, which are critical for adsorption performance. Furthermore, the activated carbon prepared from caraway seed at 800 °C exhibited a very low total surface area of just 10 m^2^/g and a total pore volume of 0.04 cm^3^/g, with an average pore size of 15.83 nm and an iodine number of only 130 mg/g [23]. The values obtained for the material under investigation are significantly lower than those of the oak bark adsorbent activated at the same temperature (452 m^2^/g surface area, 0.958 cm^3^/g pore volume, and 369 mg/g iodine number). This finding indicates that the development of porosity in the material derived from caraway has been limited. The markedly diminished iodine number is indicative of a substantially diminished microporosity and adsorption potential. This marked contrast underscores the pivotal role of precursor type in dictating the efficiency of physical activation processes, with oak bark demonstrating a markedly superior aptitude for the production of high-performance activated carbons. In general, oak bark has been shown to exhibit considerably higher activation efficiency when subjected to CO_2_ treatment, resulting in the production of an activated carbon sample with exceptional surface and adsorptive characteristics. It is evident that oak bark has the potential to be a viable raw material for the production of activated carbon through a direct activation process with carbon dioxide. The results of the study indicate that this material is suitable for the production of low-cost adsorbents with well-developed textural parameters. A comparison of sorption materials obtained from oak bark with other bark-based adsorbents described in the literature reveals that Populus euramevicana bark activated with steam at 700 and 800 °C had a specific surface area of 548 and 556 m^2^/g, respectively, and an iodine number of 456 and 499 mg/g, respectively [24]. Activated carbon obtained from the bark waste of Acacia mangium by chemical activation with 50% orthophosphoric acid (V) at 500 °C for 120 min exhibited a total surface area of 415 m^2^/g [25]. Activated carbon obtained from the bark waste of Acacia mangium by chemical activation with 50% orthophosphoric acid (V) at 500 °C for 120 min exhibited a surface area of 391 m^2^/g [26].

These comparisons demonstrate the superior performance of oak bark as a precursor for activated carbon compared to other biomass sources. Its ability to develop high surface area, significant porosity, and adsorption properties underlines its potential for efficient pollutant removal. Given its natural abundance and low cost, oak bark represents a highly promising feedstock for the scalable and sustainable production of high-quality activated carbon materials.

**Figure 3:**
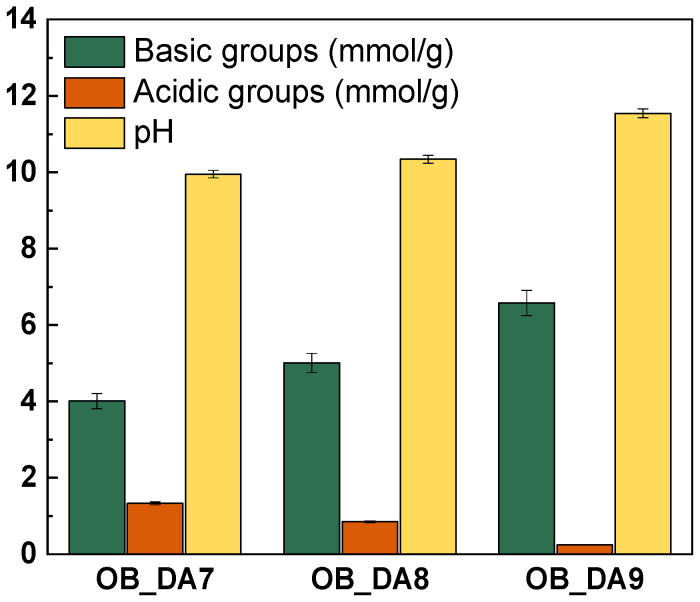
Content of oxygen functional groups on the surface of obtained adsorbents.

**Figure 4:**
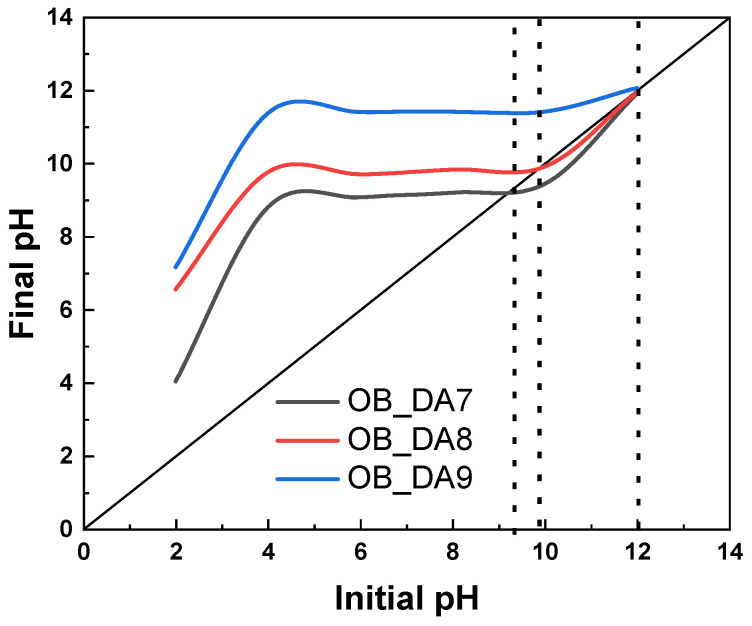
The point of zero charge (pH_pzc_) of the tested samples.

An investigation was conducted into the acid-based properties of the samples obtained, as illustrated in Figure 3. The analysis demonstrated that all activated carbon materials exhibited a predominance of basic groups, with the OB_DA7 sample displaying 4.01 mmol/g, the OB_DA8 sample exhibiting 5.01 mmol/g, and the OB_DA9 sample exhibiting 6.58 mmol/g, while the acidic groups measured 1.33 mmol/g, 0.85 mmol/g, and 11.54 mmol/g, respectively. The presence of acidic oxygen functional groups on the surface of activated carbon, resulting from its oxidation at elevated temperatures, contributes to the adsorbent’s polar and hydrophilic characteristics. In contrast, the generation of basic oxygen functional groups is attributable to oxygen chemisorption on the adsorbent surface at elevated temperatures and within an oxidizing gas atmosphere [27]. The pH_pzc_ values of the obtained samples were determined using the drift method (Figure 4).

The pH_pzc_ for the carbon sorbents tested falls between a range of 9.3 and 11.9, indicating that the materials produced possess a basic character. In accordance with the definition of the point of zero charge, the surface of the adsorbent is negatively charged when the pH is greater than the pH of the point of zero charge (pH > pH_pzc_) and positively charged when the pH is less than the pH of the point of zero charge (pH < pH_pzc_) [28]. The pH _pzc_ value increases as the activation temperature increases. This is consistent with the acid-based properties of the materials tested. The pH_pzc_ value increases when there is an increase in basic group content.

Table 3 presents the elemental composition and ash content of raw oak bark and three derived samples (OB_DA7, OB_DA8, and OB_DA9). The raw oak bark contains relatively low nitrogen (N^daf^: 0.42%) and sulfur (S^daf^: 0.14%), but a high oxygen content (O^daf*^: 45.80%), and moderate carbon (C^daf^: 47.55%) and hydrogen (H^daf^: 6.08%) contents. The derived samples demonstrated a substantial increase in carbon content, particularly OB_DA7 (83.63%), suggesting enhanced carbonization or thermal treatment. Conversely, a sharp decrease in oxygen content was observed, particularly in OB_DA7 (13.69%), indicating the removal of volatile compounds. The levels of hydrogen and nitrogen were found to be relatively low across all derived samples, while sulfur remained stable. It is noteworthy that the ash content increased from OB_DA7 (12.72%) to OB_DA9 (17.23%).

**Figure 5:**
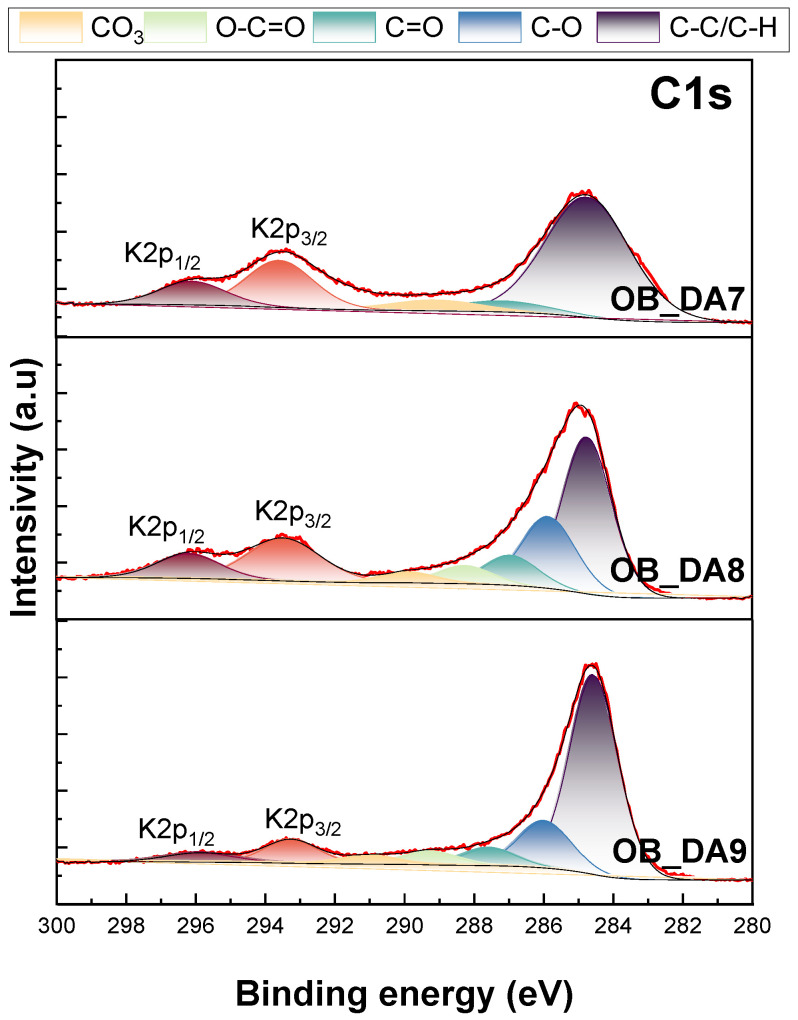
The XPS carbon (C1s) spectra of the obtained activated carbon samples.

X-ray photoelectron spectroscopy was utilized for the qualitative analysis of the functional groups present on the surfaces of the samples. The spectra of C1s are presented in Figure 5. Table 4 presents the elemental contents, expressed as percentage atomic concentration (% At). Analysis of the C1s spectra from the obtained samples revealed three to five peaks at 284.74–284.57 eV, which can be assigned to C–C/C–H carbon; at 285.84–285.97 eV, assigned to C–O groups; at 286.92–287.55 eV, assigned to C=O groups; at 288.22–289.25 eV, assigned to O-C=O; and at 289.86–290.99 eV, assigned to CO_3_ [29,30].

In the XPS spectra, peaks originating from K2p 1/2 and K2p 3/2 can be distinguished. The presence of potassium on the surface of the adsorbents tested can be attributed to the organic starting material. A comparison of the activated carbon samples obtained revealed a direct correlation between the activation temperature and the content of oxygen, with a concomitant decrease in the content of carbon and potassium (Table 4). The activation method used is the reason why there was comparatively low oxygen content on the surface of the materials [31].

### 3.2. Adsorption of Butylparaben

**Figure 6:**
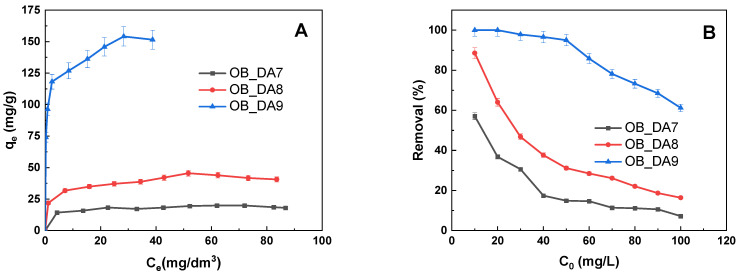
The isotherm of butylparaben adsorption on obtained activated carbon (**A**) and the correlation between the removal (%) of the pollutant solution and its initial concentration (**B**) (volume of dye solution: 0.05 L; dye concentration: 10–100 mg/L; shaking speed: 250 rpm/min; temperature: 298.15 ± 1 K).

Figure 6 illustrates the adsorption isotherms of butylparaben. It was found that the sorption capacity of the sample depended on the activation temperature. As demonstrated in Figure 6, the sample obtained at 900 °C exhibited markedly superior sorption capacity in comparison to those obtained at lower temperatures. The maximum sorption capacities of the materials obtained for butylparaben are as follows: 154 mg/g for the OB_DA9 sample, 46 mg/g for OB_DA8, and 20 mg/g for OB_DA7, respectively. The results obtained in this study are consistent with the specific surface area of the adsorbents that were tested.

A comparison of the results obtained for the adsorption of butylparaben on the carbon materials tested with those reported in the literature revealed that the sorption capacities obtained for samples OB_DA8 and OB_DA9 far exceeded those of other adsorbents (Table 5). The sorption capacity of biochar derived from fique bagasse, which had been obtained by activation with NaOH at 800 °C, was found to be 21 mg/g [32]. In the other study, activated carbon obtained from coconut shell by physical activation with carbon dioxide at 800 °C was found to have a sorption capacity for butylparaben of 7.5 mg/g [20]. It is worthy to note that activated carbon obtained by subjecting African palm shells (subjected to physical activation with carbon dioxide at a temperature of 900 °C for 6 h) were found to be capable of adsorbing 144 mg/g [19]. However, this value is lower than that obtained for the OB_DA9 sample by 10 mg/g. It is important to note that the production cost of African palm shells’ adsorbent is higher than the OB_DA9 sample, due to the longer activation time required.

**Figure 7:**
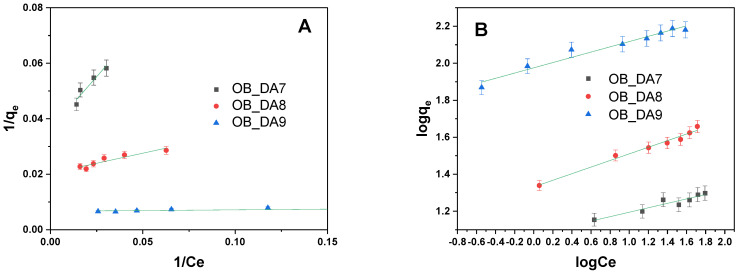
Linear fitting for butylparaben on obtained activated carbon using (**A**) a Langmuir model and (**B**) a Freundlich model.

Correlations between adsorption experimental data and theoretical or empirical isothermal models were established. These correlations yield significant insights into the mechanisms of interaction between the dye and the sorbent, thereby facilitating a more profound comprehension of the adsorption process in its entirety. The adsorption behavior of butylparaben onto obtained samples was analyzed using both Langmuir and Freundlich isotherm models (Table 6). As illustrated in Figure 7, the data obtained were subjected to linear regression analysis in order to generate a fit with the models under consideration.

The analysis of the data indicated that the Freundlich model exhibited the optimal correspondence to the data. The Freundlich constant (K_F_) exhibited a substantial increase from OB_DA7 (11.877 mg/g(L/mg)^1/n^) to OB_DA9 (94.497 mg/g(L/mg)^1/n^), thereby providing further evidence to support the hypothesis that enhanced adsorption capacity has been achieved. The low 1/n values (<0.5) observed across all samples are indicative of favorable adsorption [33]. These results suggest that the adsorption of butylparaben is better described by the Freundlich model, particularly for high-capacity sorbents like OB_DA9, thereby highlighting the heterogeneous and multilayer nature of the adsorption process.

**Figure 8:**
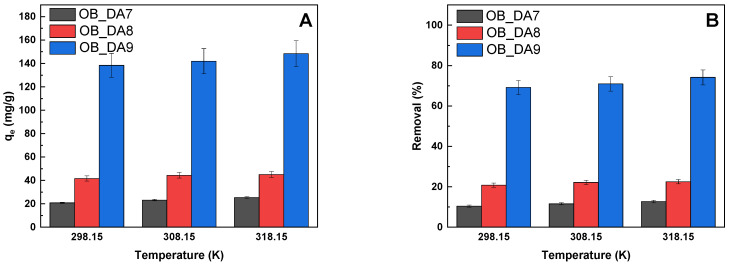
The effect of temperature of the aqueous solution of butylparaben on (**A**) sorption capacities (mg/g) and (**B**) removal (%) (volume of butylparaben solution: 0.05 L; butylparaben concentration: 80 mg/L for all samples; shaking speed: 250 rpm/min,).

Figure 8 demonstrates the correlation between the sorption capacities and percentage removal of butylparaben, as influenced by the temperature of the adsorption process. A thorough examination of the results obtained revealed that temperature exerts a negligible influence on the adsorption process. This outcome is economically advantageous, as it leads to a reduction in the cost of the adsorption process.

An analysis of the thermodynamic parameters for the adsorption of butylparaben onto the sorbent samples provided insights into the spontaneity, heat changes, and disorder that are associated with the process (Table 7). For OB_DA7 and OB_DA8, the ∆G^0^ values were positive across all temperatures, indicating a non-spontaneous adsorption process under the studied conditions. In contrast, OB_DA9 exhibited negative ∆G^0^ values that became more negative with increasing temperature (from −4.27 to −5.21 kJ/mol), suggesting that the adsorption is spontaneous and becomes more favorable at higher temperatures. The enthalpy changes (∆H^0^) were positive for all samples, indicating that the adsorption of butylparaben is endothermic in nature. Notably, OB_DA9 showed the highest ∆H^0^ (9.72 kJ/mol), implying greater heat absorption during adsorption. The entropy change (∆S^0^) was also highest for OB_DA9 (46.837 J/mol·K), indicating a substantial increase in randomness at the solid–liquid interface during the process of adsorption. These results highlight OB_DA9 as the most thermodynamically favorable and efficient sorbent for butylparaben adsorption among the three samples.

**Figure 9:**
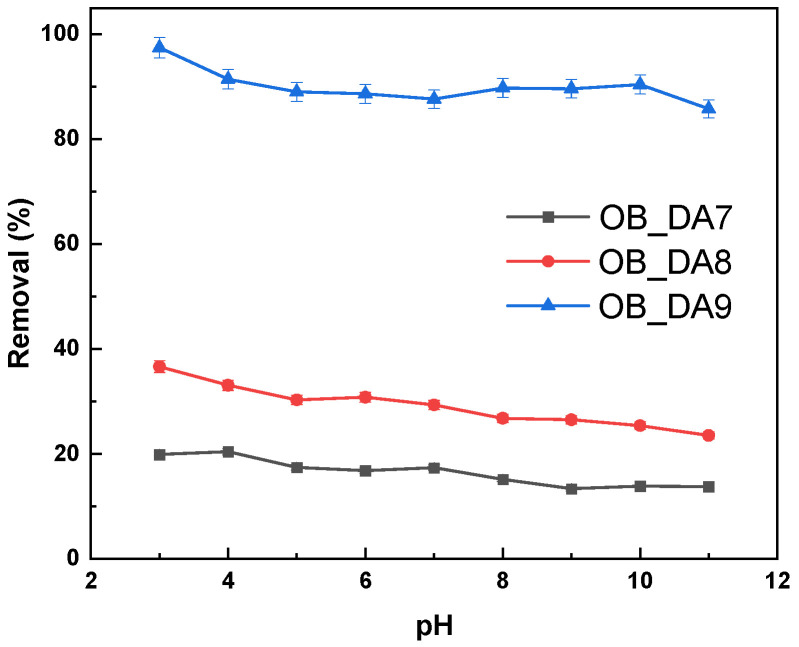
Influence of the pH of the aqueous solution of butylparaben on removal (volume of butylparaben solution: 0.05 L; butylparaben concentration: 80 mg/L for all samples; shaking speed: 250 rpm/min; temperature: 298.15 ± 1 K).

Figure 9 illustrates the effect of pH on the removal efficiency of butylparaben by three samples. Across the pH range of 3 to 11, all three samples show a gradual decline in removal efficiency as pH increases, with OB_DA9 consistently demonstrating the highest performance (above 85% of removal), followed by OB_DA8 (24–37%) and OB_DA7 (14–20%). This trend indicates that the adsorption of butylparaben is more favorable under acidic conditions and becomes less efficient at higher pH levels. Butylparaben (butyl 4–hydroxybenzoate) is a weakly acidic compound, with a pK_a_ around 8.4 [34]. Below this pH, it primarily exists in its neutral molecular form, which interacts more readily with the hydrophobic surfaces of activated carbons via van der Waals forces and π–π interactions [35]. As the pH increases beyond the pK_a_, butylparaben becomes deprotonated (anionic form), reducing its affinity for the largely nonpolar carbon surfaces due to increased electrostatic repulsion and reduced hydrophobicity. Therefore, the observed decline in adsorption capacity with rising pH can be attributed to the shift in the ionization state of butylparaben.

**Figure 10:**
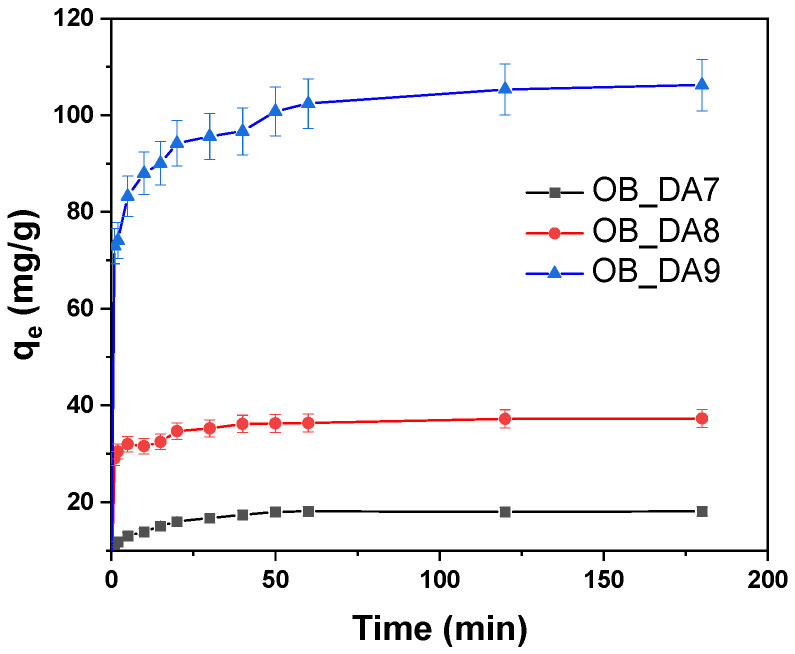
The influence of contact time on butylparaben removal (volume of butylparaben solution: 0.05 L; butylparaben concentration: 80 mg/L for all samples; shaking speed: 250 rpm/min; temperature: 298.15 ± 1 K).

The effect of contact time between the adsorbent and the adsorbate on the efficiency of pollutant removal by the obtained activated carbons was investigated. As illustrated in Figure 10, it can be deducted that the adsorption equilibrium is achieved after approximately 60 min. From an economic perspective, this process is highly advantageous due to its expeditious nature. The results obtained were then utilized to ascertain the kinetic model of adsorption on the activated carbons that had been obtained (Table 8).

**Figure 11:**
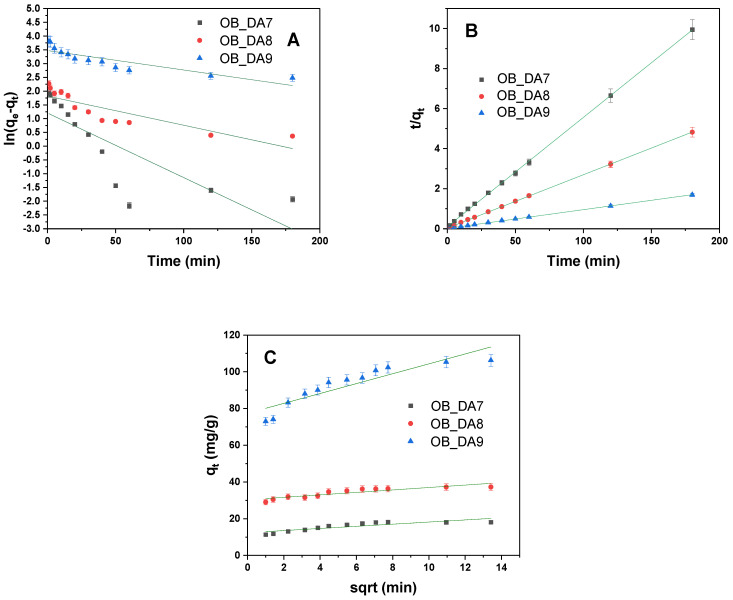
Linear fitting to (**A**) pseudo-first-order model, (**B**) pseudo-second-order model, and (**C**) intraparticle diffusion model.

The kinetic parameters for the adsorption of butylparaben onto activated carbon samples were evaluated using pseudo-first-order, pseudo-second-order, and intraparticle diffusion models. The results of this evaluation are summarized in Table 8. As demonstrated in Figure 11, the data have been fitted to the selected kinetic models using linear regression.

The pseudo-second-order model exhibited the best fit for all samples, with exceptionally high R^2^ and adjusted R^2^ values (0.999) and calculated equilibrium adsorption capacities (q_e/cal_) closely matching the experimental values (q_e_). In contrast, the pseudo-first-order mode showed poor correlation, with significantly lower R^2^ values (0.662–0.751) and underestimated q_e/cal_ values, indicating that it does not accurately describe the adsorption kinetics of butylparaben on these materials. The intraparticle diffusion model showed a moderate fit (R^2^ = 0.744–0.825), suggesting that, while diffusion within pores may play a role, it is not the sole rate-limiting step. Overall, these findings indicate that chemisorption, as described by the pseudo-second-order model, is the dominant mechanism governing the adsorption of butylparaben onto the tested activated carbons.

### 3.3. Adsorption of Methylene Blue

**Figure 12:**
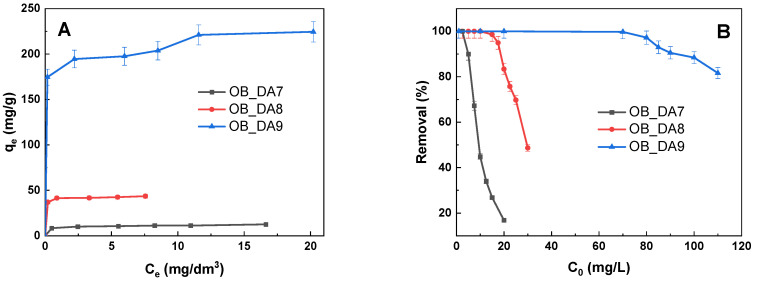
Isotherm of methylene blue adsorption on obtained activated carbon (**A**) and correlation between the removal (%) of the pollutant solution and its initial concentration (**B**) (volume of dye solution: 0.05 L; dye concentration: 5–110 mg/L; shaking speed: 250 rpm/min; temperature: 298.15 ± 1 K).

Figure 12 presents the adsorption isotherms of methylene blue. Similarly to the adsorption of butylparaben, the sorption capacity of the sample was found to depend on the activation temperature. As demonstrated in Figure 12, the sample obtained at 900 °C had a significantly higher sorption capacity than those obtained at lower temperatures. The maximum sorption capacities of the materials obtained for methylene blue are as follows: 224 mg/g for OB_DA9, 44 mg/g for OB_DA8, and 13 mg/g for OB_DA7.

A comparison can be drawn between the sorption capacities obtained for methylene blue and the values obtained for other carbon adsorbents (Table 9). The sorption capacity of sludge-derived biochar, which had been subjected to physical activation, for methylene blue was only 24 mg/g [36]. In contrast, biochar obtained from barley malt bagasse through physical activation with carbon dioxide exhibited a sorption capacity for methylene blue of 161 mg/g [37]. Furthermore, it was determined that the commercial activated carbon Norit^®^ SX2 exhibited a sorption capacity of 161.3 mg/g, which is less than that observed for the sample of OB_DA, at a rate of almost 63 mg/g [38].

It has been demonstrated that one gram of activated carbon obtained by the KOH chemical activation of baobab fruit shell has the capacity to adsorb 114 mg of methylene blue [39]. It is noteworthy that the sample designated OB_DA9, which exhibited a lower cost direct activation with CO_2_, and it demonstrated a sorption capacity for the dye that was approximately 100–110 mg/g higher than that of the samples obtained by chemical activation with KOH. In summary, studies of the sorption capacities of activated carbons obtained by the direct activation of oak bark demonstrated that adsorbents obtained at 700 and 800 °C exhibited average sorption capacities. However, it was observed that the sample OB_DA9, activated at the highest temperature, exhibited the capacity to adsorb a quantity of the tested dye that exceeds the results obtained for commercial and chemically activated carbons.

**Figure 13:**
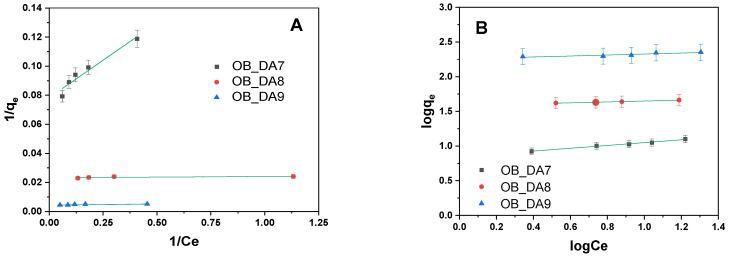
Linear fitting for methylene blue on obtained activated carbon using (**A**) a Langmuir model and (**B**) a Freundlich model.

Establishing a correlation between the experimental adsorption data and theoretical or empirical isothermal models offers valuable insight into the interaction mechanisms between the dye and the sorbent, thereby enhancing the overall understanding of the adsorption process. As illustrated in Figure 13, the experimental results show a strong linear fit with both the Langmuir and Freundlich isotherm models. To assess the suitability of these models, their respective parameters were determined and are presented in Table 10. The adsorption behaviors of methylene blue onto activated carbon samples were evaluated using Langmuir and Freundlich isotherm models. The experimental adsorption capacity (q_exp_) exhibited a marked increase with increasing pyrolysis temperature, rising from 13 mg/g for the OB_DA7 to 224 mg/g for the OB_DA9. This trend suggests that elevated pyrolysis temperatures enhance the sorption properties of activated carbon through augmented surface area and porosity, thereby facilitating enhanced dye uptake. The Langmuir isotherm model, which assumes monolayer adsorption on a homogeneous surface, provided a satisfactory fit for all samples, particularly for the OB_DA9 activated carbon. This sample exhibited a maximum monolayer adsorption capacity (q_max_) of 229 mg/g, matching the experimental value (224 mg/g), with an R^2^ of 0.999 and an Adj^2^ of 0.999, indicating near-perfect model conformity.

The Langmuir constant (K_L_), which reflects the binding sites’ affinity, was observed to be highest for the OB_DA8 sample (3.758 L/mg) [40]. This finding suggests that this temperature yields surface characteristics that are conducive to favorable dye interactions. In contrast, the Freundlich isotherm model, which accounts for heterogeneous surface adsorption and multilayer formation, demonstrated superior performance for the OB_DA 7 and OB_DA8 samples, with R^2^ values of 0.989 and 0.985, respectively. The Freundlich constant (K_F_) and the adsorption intensity parameter (1/n) provide further evidence to support the hypothesis that favorable adsorption conditions are in place across all temperatures, particularly at 800°C and 900 °C, where 1/n values were found to be notably low (0.065 and 0.071, respectively), indicating strong adsorption affinity [41]. However, for the OB_DA9 sample, the Freundlich model fit deteriorated (R^2^ = 0.798), suggesting a transition toward more uniform adsorption sites and a predominance of monolayer coverage, as better described by the Langmuir model. In summary, the isotherm analysis demonstrated that an increase in pyrolysis temperature results in enhanced adsorption capacity.

**Figure 14:**
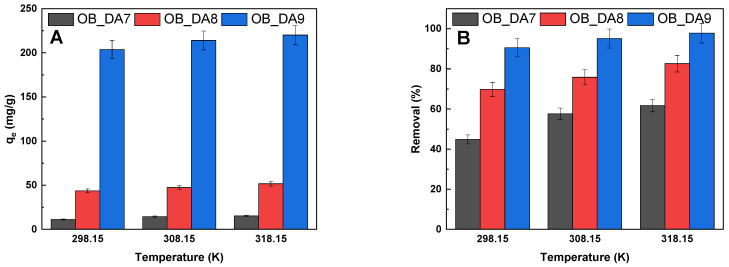
Impact of temperature of the aqueous solution of dye on (**A**) sorption capacities (mg/g) and (**B**) removal (%) (volume of dye solution: 0.05 L; dye concentration: 10 mg/L for OB_DA7 sample, 25 mg/L for OB_DA8 sample, and 90 mg/L for OB_DA9 sample; shaking speed: 250 rpm/min, temperature: 298.15 ± 1 K).

The thermodynamic analysis provides valuable insights into whether the adsorption process involves physisorption or chemisorption. As temperature increases, added heat is converted into kinetic energy, enhancing the mobility of dye molecules and their interaction with the adsorbent surface. To evaluate the thermodynamic behavior, experiments were carried out at three temperatures of 298.15 K, 308.15 K, and 318.15 K. As demonstrated in Figure 14, there was a general increase in both dye removal efficiency and adsorption capacity with rising temperature. The specific values for this increase are given in 298.15 K, 308.15 K, and 318.15 K. Nonetheless, the overall enhancement in sorption capacity for methylene blue was relatively modest, ranging from 7.28% to 16.82% between 298.15 K and 318.15 K. From a pragmatic and economic standpoint, the process remains advantageous since it functions efficiently at room temperature without necessitating additional energy input.

Table 11 summarizes the theoretical thermodynamic parameters calculated for the adsorption of methylene blue onto different adsorbent samples. The enthalpy change (ΔH^0^) values for all samples were positive, indicating that the adsorption process is endothermic. Specifically, ΔH^0^ values for the samples OB_DA7, OB_DA8, and OB_DA9 were 27.01 kJ/mol, 28.35 kJ/mol, and 61.01 kJ/mol, respectively. These moderate ΔH^0^ values suggest that the adsorption mechanism is likely physisorption [42]. Gibbs free energy change (ΔG^0^) was negative across all samples and temperatures, confirming that the adsorption process is spontaneous [42]. Entropy change (ΔS^0^), which reflects the degree of disorder during adsorption, increases with higher-temperature samples. The OB_DA9 sample exhibited a ΔS^0^ of 230.90 J/mol × K, significantly higher than that of OB_DA8 (109.546 J/mol × K) and OB_DA7 (96.978 J/mol × K). This suggests that adsorption on OB_DA9 introduces a greater degree of randomness, likely due to more active adsorption sites or structural characteristics of the material [42].

**Figure 15:**
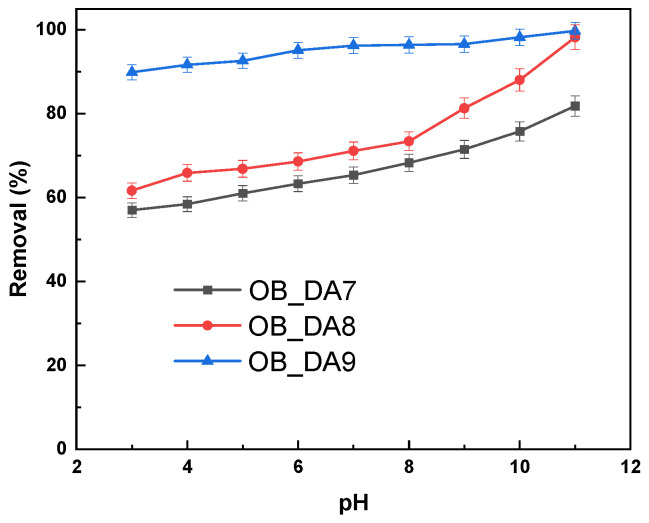
Influence of pH of the aqueous solution of dye on removal (volume of dye solution: 0.05 L; dye concentration: 10 mg/L for OB_DA7 sample, 25 mg/L for OB_DA8 sample, and 90 mg/L for OB_DA9 sample; shaking speed: 250 rpm/min; temperature: 298.15 ± 1 K).

The effect of pH on the removal efficiency of methylene blue by the produced activated carbon was evaluated, as demonstrated in Figure 15. The findings indicate that an increase in the pH of the dye solution results in an enhancement of the adsorption capacity of both activated carbon samples. It is noteworthy that both adsorbents exhibited a substantial decline in sorption capacity at a pH of 3, suggesting that acidic conditions are less conducive to dye uptake. At elevated pH levels, the surface of the activated carbon became negatively charged, thereby facilitating the adsorption of positively charged methylene blue molecules. This phenomenon is consistent with the findings of previous studies. Moreover, the findings suggest that the optimal pH range for methylene blue adsorption is in the range of 6 and 11, thereby corroborating the current observations pertaining to activated carbon derived from oak bark [43].

**Figure 16:**
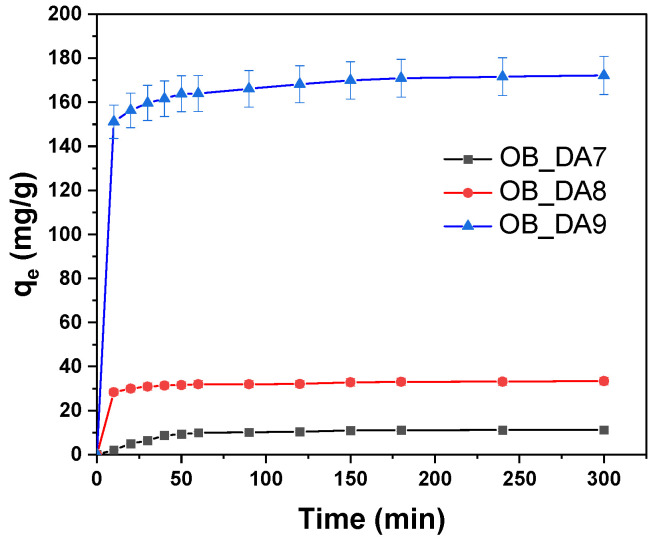
Impact of contact time on methylene blue removal (volume of dye solution: 0.05 L; dye concentration: 10 mg/L for OB_DA7 sample, 25 mg/L for OB_DA8 sample, and 90 mg/L for OB_DA9 sample; shaking speed: 250 rpm/min; temperature: 298.15 ± 1 K).

The influence of contact time on the dye removal efficiency of the tested activated carbons was evaluated to determine the duration required to reach adsorption equilibrium. As depicted in Figure 16, equilibrium was established within approximately 60 to 90 min, highlighting the rapid adsorption kinetics of the materials. This swift equilibrium time is particularly beneficial from an economic standpoint, as it suggests reduced operational time and energy consumption.

**Figure 17:**
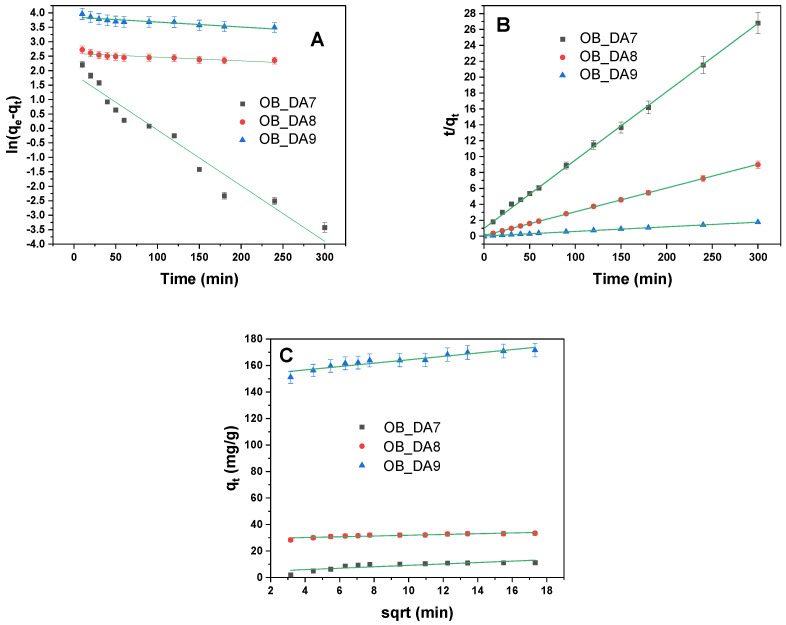
Linear fitting to (**A**) pseudo-first-order model, (**B**) pseudo-second-order model, and (**C**) intraparticle diffusion model.

The adsorption kinetics of the three samples—OB_DA7, OB_DA8, and OB_DA9—were analyzed using three kinetic models (Table 12). The corresponding graphs for the linear kinetic models are displayed in Figure 17. The pseudo-first-order model demonstrated an inadequate fit, particularly for OB_DA8 and OB_DA9, exhibiting low calculated q_e_ values and R^2^ values below 0.83. In contrast, the pseudo-second-order model demonstrated an excellent fit for all samples, with R^2^ values close to 1 and predicted q_e_ values nearly identical to the experimental data, suggesting that chemisorption is the primary mechanism governing the adsorption process [43].

The intraparticle diffusion model indicated a certain degree of contribution to the overall kinetics, particularly for OB_DA9, which exhibited the highest diffusion rate constant (k_id_) and boundary layer thickness (C) [43]. However, the R^2^ values for this model were lower, indicating that intraparticle diffusion is not the sole rate-limiting step. In conclusion, the data demonstrate that the adsorption kinetics are most adequately described by the pseudo-second-order model, thus emphasizing the significance of chemical interactions in the process.

### 3.4. Adsorption Mechanism

The adsorption mechanism of the prepared activated carbon materials can be understood through their surface chemistry and electronic interactions, as supported by Boehm titration and XPS analysis. Boehm titration indicates a predominance of basic functional groups and a basic surface pH, with pH_p_zc values ranging from 9.3 to 11.9, suggesting that the carbon surfaces are primarily basic in nature. Such characteristics favor the adsorption of acidic or positively charged pollutants through electrostatic interactions. XPS analysis of the C1s spectra reveals the presence of oxygen-containing groups such as C–O, C=O, O–C=O, and CO_3_, which contribute to the adsorption of polar molecules via hydrogen bonding or dipole interactions. In addition, the aromatic C–C/C–H functionalities support π–π interactions with aromatic pollutants like methylene blue and butylparaben.

XPS data also indicate a significant surface potassium content, with levels reaching up to 18.91% (Table 4). Potassium plays a dual role; it may catalyze pore formation during activation, thereby enhancing surface area and porosity, and it can also facilitate electrostatic adsorption by creating localized negative sites that attract cationic pollutants like methylene blue. Despite originating as a by-product of the precursor or activation process, potassium significantly influences the textural and chemical properties of the adsorbent and must be considered in interpreting adsorption performance [44].

The differing adsorption behaviors of these two compounds can be attributed to their molecular characteristics. Methylene blue, a cationic dye, interacts primarily through electrostatic attraction and ion exchange with negatively charged sites, favoring homogeneous adsorption and aligning well with the Langmuir model. In contrast, butylparaben (BuP), a neutral and hydrophobic molecule, interacts mainly through π–π stacking and van der Waals forces on heterogeneous surfaces, which is better described by the Freundlich model [45].

Kinetic modeling further supports these interpretations. The adsorption of both pollutants follows a pseudo-second-order model, indicating that chemisorption is the dominant mechanism. For methylene blue, electrostatic attraction dominates, while, for butylparaben, π–π interaction and hydrogen bonding are more relevant. The intraparticle diffusion model also suggests that, while pore diffusion contributes to the overall process—especially in materials with higher surface areas—it is not the sole rate-limiting step. This multi-step behavior confirms that chemical interactions and surface heterogeneity are key factors controlling the adsorption process.

## 4. Conclusions

The results obtained indicate that oak bark can successfully be employed as a precursor for activated carbon samples obtained by direct activation, for the removal of methylene blue and butylparaben from aqueous solutions. Physicochemical characterization revealed that the activation temperature significantly influences the specific surface area and sorption properties of the samples. The specific surface area of the materials obtained was 247 m^2^/g for the sample obtained at 700 °C, 452 m^2^/g for the sample obtained at 800 °C, and 696 m^2^/g for the sample obtained at 900 °C. XPS analysis revealed the presence of carbon, potassium, and oxygen on the surface of the adsorbents. The surface of the samples exhibited a higher content of basic than acidic groups. The most effective sorption properties were demonstrated by activated carbon produced at a temperature of 900 °C. The material exhibited a maximum sorption capacity of 154 mg/g for butylparaben and 224 mg/g for methylene blue. The differences in adsorption isotherms between methylene blue and butylparaben likely arise from their distinct molecular properties and interaction mechanisms with the carbon surface. While methylene blue shows more uniform adsorption behavior, consistent with the Langmuir model, butylparaben interacts through weaker, non-specific forces, aligning better with the Freundlich model. The results showed that the sorption capacity of the tested activated carbon materials increased with rising pH in the case of methylene blue, whereas, for butylparaben, an increase in pH led to a decrease in sorption capacity. The subsequent phase of the research will focus on the adjustment of activation parameters, including gas flow rate and activation time, with the objective of minimizing overall process costs. This will include the investigation of the desorption process, in addition to the testing of the sorption capacity of the adsorbents on actual wastewater.

## Figures and Tables

**Table 1:** Information about the chosen contaminants.

Dye	Chemical Formula	Structure	Mass (g/mol)	λ_max_ (nm)
**Methylene blue**	[C_16_H_18_N_3_S] + Cl^−^	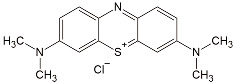	319.85	665
**Butylparaben**	C_11_H_14_O_3_	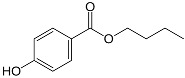	194.23	254

**Table 2:** Textural parameters, iodine number, and yield of the obtained carbon sorbent samples.

Sample	Surface Area (m^2^/g)		Pore Volume (cm^3^/g)		Average Pore Size (nm)	Iodine Number (mg/g)	Yield (%)
	Total	Micropore	Total	Micropore			
**OB_DA7**	247	152	0.480	0.086	3.80	277	49.24
**OB_DA8**	452	198	0.958	0.107	4.81	369	43.52
**OB_DA9**	696	141	1.445	0.077	6.35	741	38.91

**Table 3:** Elemental composition and content of ash of the obtained adsorbents (wt.%).

Sample	N^daf^	C^daf^	H^daf^	S^daf^	O^daf*^	% Ash
**Oak bark**	0.42	47.55	6.08	0.14	45.80	1.40
**OB_DA7**	1.59	83.63	0.31	0.78	13.69	12.72
**OB_DA8**	1.72	80.17	0.32	0.80	17.00	15.58
**OB_DA9**	1.71	73.65	0.27	0.80	23.57	17.23

*—By difference; method error ≤ 0.3%.

**Table 4:** Relative contents of elements (% At) for the obtained samples based on XPS analysis.

Element	OB_DA7	OB_DA8	OB_DA9
**O**	6.02	8.02	10.79
**C**	75.07	73.59	71.31
**K**	18.91	18.39	17.90

**Table 5:** Comparison of the activated carbons obtained with various reported adsorbent and their sorption capacities for butylparaben.

Precursor	Activator	Activation Time (min)	Activation Temperature (°C)	Sorption Capacity (mg/g)	Source
**Oak bark**	CO_2_	60	700	20	This study
	CO_2_	60	800	46	
	CO_2_	60	900	154	
**Fique bagasse**	NaOH	120	800	21	[32]
**Coconut shell**	CO_2_	180	800	7.5	[20]
**African palm shells**	CO_2_	360	900	144	[19]

**Table 6:** The values of constants determined for the linear Langmuir and Freundlich models for the experimental data of butylparaben.

Model	Parameters	Sample		
		OB_DA7	OB_DA8	OB_DA9
	**q_exp_ (mg/g)**	20	46	154
**Langmuir**	**K_L_ (L/mg)**	0.048	0.140	0.047
	**q_m_ (mg/g)**	28	49	150
	**R^2^**	0.909	0.864	0.749
	**Adj^2^**	0.864	0.830	0.686
**Freundlich**	**K_F_ (mg/g(L/mg)^1/n^)**	11.877	16.658	94.497
	**1/n**	0.120	0.179	0.142
	**R^2^**	0.911	0.985	0.962
	**Adj^2^**	0.893	0.982	0.955

**Table 7:** Thermodynamic parameters of butylparaben adsorption on the obtained sorbents.

Sample	Temperature (K)	∆G^0^ (kJ/mol)	∆H^0^ (kJ/mol)	∆S^0^ (J/mol × K)
**OB_DA7**	298.15	3.06	8.82	19.351
	308.15	2.86		
	318.15	2.67		
**OB_DA8**	298.15	1.04	3.97	9.899
	308.15	0.87		
	318.15	0.84		
**OB_DA9**	298.15	–4.27	9.72	46.837
	308.15	–4.63		
	318.15	–5.21		

**Table 8:** The values of constants for kinetic models of butylparaben adsorption.

Model	Parameters	Sample		
		OB_DA7	OB_DA8	OB_DA9
	**q_e_ (mg/g)**	18	37	111
**Pseudo-first-order**	**k_1_ (1/min)**	1.63 × 10^−6^	7.36 × 10^−6^	4.93 × 10^−6^
	**q_e/cal_ (mg/g)**	3	6	32
	**R^2^**	0.662	0.747	0.751
	**Adj^2^**	0.643	0.724	0.739
**Pseudo-second-order**	**k_2_ (g/mg × min)**	2.7 × 10^−2^	1.8 × 10^−2^	4.0 × 10^−3^
	**q_e/cal_ (mg/g)**	18	38	108
	**R^2^**	0.999	0.999	0.999
	**Adj^2^**	0.999	0.999	0.999
**Intraparticle diffusion**	**k_id_ (mg/g × min^1/2^** **)**	0.58	0.67	0.58
	**C (mg/g)**	12	30	77
	**R^2^**	0.744	0.797	0.825
	**Adj^2^**	0.719	0.776	0.807

**Table 9:** Comparison of the activated carbons obtained with various reported adsorbent and their sorption capacities for methylene blue.

Precursor	Activator	Activation Time (min)	Activation Temperature (°C)	Sorption Capacity (mg/g)	Source
**Oak bark**	CO_2_	60	700	13	This study
	CO_2_	60	800	44	
	CO_2_	60	900	224	
**Sludge**	-	120	550	24	[36]
**Barley malt bagasse**	CO_2_	60	800	161	[37]
**Baobab fruit shell**	KOH	60	500	114	[39]

**Table 10:** The values of constants determined for the linear Langmuir and Freundlich models for the experimental data of methylene blue.

Model	Parameters	Sample		
		OB_DA7	OB_DA8	OB_DA9
	**q_exp_ (mg/g)**	13	44	224
**Langmuir**	**K_L_ (L/mg)**	0.762	3.758	2.586
	**q_max_ (mg/g)**	13	45	229
	**R^2^**	0.945	0.943	0.999
	**Adj^2^**	0.927	0.886	0.999
**Freundlich**	**K_F_ (mg/g(L/mg)^1/n^)**	7.011	38.367	180.090
	**1/n**	0.203	0.065	0.071
	**R^2^**	0.989	0.985	0.798
	**Adj^2^**	0.985	0.978	0.731

**Table 11:** Thermodynamic parameters of methylene blue adsorption on the obtained activated carbon.

Sample	Temperature (K)	∆G_0_ (kJ/mol)	∆H_0_ (kJ/mol)	∆S_0_ (J/mol × K)
**OB_DA7**	298.15	−1.76	27.01	96.98
	308.15	−3.13		
	318.15	−3.68		
**OB_DA8**	298.15	−4.34	28.35	109.55
	308.15	−5.28		
	318.15	−6.54		
**OB_DA9**	298.15	−7.87	61.01	230.90
	308.15	−9.96		
	318.15	−12.50		

**Table 12:** The values of constants determined for kinetic models for methylene blue adsorption.

Model	Parameters	Sample		
		OB_DA7	OB_DA8	OB_DA9
	**q_e_ (mg/g)**	11	44	204
**Pseudo-first-order**	**k_1_ (1/min)**	8.02 × 10^−5^	5.38 × 10^−6^	7.17 × 10^−6^
	**q_e/cal_ (mg/g)**	6.50	13	47
	**R^2^**	0.952	0.721	0.829
	**Adj^2^**	0.945	0.690	0.810
**Pseudo-second-order**	**k_2_ (g/mg × min)**	3.76 × 10^−3^	1.66 × 10^−2^	2.19 × 10^−2^
	**q_e/cal_ (mg/g)**	12	43	199
	**R^2^**	0.998	0.999	0.999
	**Adj^2^**	0.998	0.999	0.999
**Intraparticle diffusion**	**k_id_ (mg/g × min^1/2^** **)**	0.53	0.23	1.28
	**C (mg/g)**	3.83	29.00	152
	**R^2^**	0.683	0.809	0.893
	**Adj^2^**	0.652	0.790	0.883

## Data Availability

The original contributions presented in this study are included in the article. Further inquiries can be directed to the corresponding author.
